# Monolithically Integrated Diffused Silicon Two-Zone Heaters for Silicon-Pyrex Glass Microreactors for Production of Nanoparticles: Heat Exchange Aspects

**DOI:** 10.3390/mi11090818

**Published:** 2020-08-28

**Authors:** Milena Rašljić Rafajilović, Katarina Radulović, Milče M. Smiljanić, Žarko Lazić, Zoran Jakšić, Dragomir Stanisavljev, Dana Vasiljević Radović

**Affiliations:** 1 Center of Microelectronic Technologies, Institute of Chemistry, Technology and Metallurgy—National Institute of the Republic of Serbia, University of Belgrade, Njegoševa 12, 11000 Belgrade, Serbia; kacar@nanosys.ihtm.bg.ac.rs (K.R.); smilce@nanosys.ihtm.bg.ac.rs (M.M.S.); zlazic@nanosys.ihtm.bg.ac.rs (Ž.L.); jaksa@nanosys.ihtm.bg.ac.rs (Z.J.); dana@nanosys.ihtm.bg.ac.rs (D.V.R.); 2 Faculty of Physical Chemistry, University of Belgrade, Studentski trg 12-16, 11000 Beograd, Serbia; dragisa@ffh.bg.ac.rs

**Keywords:** high-temperature microreactors, nanoparticle synthesis, titania, titanium dioxide, silicon micromachining, Pyrex glass micromachining, integrated heater, diffusion, thermal vision

## Abstract

We present the design, simulation, fabrication and characterization of monolithically integrated high resistivity p-type boron-diffused silicon two-zone heaters in a model high temperature microreactor intended for nanoparticle fabrication. We used a finite element method for simulations of the heaters’ operation and performance. Our experimental model reactor structure consisted of a silicon wafer anodically bonded to a Pyrex glass wafer with an isotropically etched serpentine microchannels network. We fabricated two separate spiral heaters with different temperatures, mutually thermally isolated by barrier apertures etched throughout the silicon wafer. The heaters were characterized by electric measurements and by infrared thermal vision. The obtained results show that our proposed procedure for the heater fabrication is robust, stable and controllable, with a decreased sensitivity to random variations of fabrication process parameters. Compared to metallic or polysilicon heaters typically integrated into microreactors, our approach offers improved control over heater characteristics through adjustment of the Boron doping level and profile. Our microreactor is intended to produce titanium dioxide nanoparticles, but it could be also used to fabricate nanoparticles in different materials as well, with various parameters and geometries. Our method can be generally applied to other high-temperature microsystems.

## 1. Introduction

Microreactors are microfluidic systems in which chemical reactions are performed in a controlled manner within a network of microchannels whose widths and depths range from tens to hundreds of micrometers. At such dimensions, the fluid flow is laminar, while mass and heat transfer are strongly enhanced [1]. Microreactors have applications in chemical synthesis, biotechnology, analytical chemistry, photochemistry, etc. [2,3,4,5,6]. An especially important application field is nanoparticle synthesis [7]. Microreactors provide improved control over the size, distribution and shape of the produced nanoparticles due to the fine tunability of the reaction conditions. In addition, they are very suitable for the production of dangerous substances (e.g., toxins, explosives), because the hazards are reduced to the minimum with the use of minuscule quantities. They are also used for the synthesis of expensive materials [8]. Microreactors can be integrated with micromixer chambers, analysis systems, sensors and actuators to provide advanced devices such as labs on a chip [9,10,11,12,13,14,15,16].

Microreactors can be fabricated in different materials such as silicon, metals including stainless steel, glass, polymers, ceramics, etc. [17,18,19,20]. Among the most frequently used and best known technological approaches to their fabrication is anodic bonding of micromachined Pyrex glass and silicon [21]. The first Pyrex–silicon microreactors started appearing about 20 years ago [22]. Due to multitudinous advantages of this type of microreactors, even today this type of microreactor remains utilized and further improved [23]. We also chose this method, because single crystalline silicon exhibits excellent mechanical and electrical properties, has a good thermal conductivity and a low thermal expansion coefficient [24,25]. Pyrex glass has good chemical stability and transparency over a wide range of optical wavelengths, making it a good candidate for e.g., photocatalytic microreactors. Since its thermal expansion coefficient is approximately equal to that of silicon, it is used for the process of anodic bonding with silicon. As far as the serpentine microchannel structures are concerned, this approach is well established and almost as old as the MEMS microreactors themselves, while the knowledge base about them is actually even larger than that on Si–Pyrex bonding [26].

Temperature is one of the most important reaction parameters in nanoparticle synthesis, because temperature variations cause the changes of the shape and size of nanoparticles [27], and heat transfer control at microscale is quite challenging. Our focus here is on the heat exchange aspects of our model microreactor structure with two separate monolithically integrated diffused heaters.

The high pressure, high temperature microreactors are being used for a wide variety of purposes. These include, among others, nanoparticle synthesis (a large body of literature on this topic includes [28,29] and many more). Among a vast number of different nanoparticle materials, we chose to investigate the synthesis of titania (TiO_2_) nanoparticles. Titanium dioxide is widely used as a photocatalyst for solar conversion and in environmental applications [30,31,32]. Its nanoparticles can be synthesized in a great variety of ways, including the top-down approach (micro or nanomilling) and numerous bottom-up methods (e.g., sol-gel process, various microfluidic chemical processes, electrochemical synthesis, metal organic chemical vapor deposition, aerosol synthesis, microemulsion methods, etc. [8]) The synthesis of TiO_2_ nanoparticles in microreactors takes place at elevated temperatures. Different types of heaters, made of different materials and with different designs, are met in literature [33,34,35,36,37,38]. Numerous types of high temperature microreactors have been reported so far.

Among the more recent examples of high temperature microreactors was presented by Chan and coworkers [39]. Their device was fabricated from glass and utilized continuous flow. It was used for the synthesis of CdSe nanoparticles.

Microreactors made of silicon and Pyrex glass were investigated in [27,40,41]. Compared to capillary microreactors, these reactors offer a higher flexibility regarding the fabrication of different forms of microchannels. Yen and coauthors [40] designed a microreactor with a droplet flow for the synthesis of CdSe nanoparticles. This microreactor had a heating and a cooling zone, mutually isolated, using two phases (gas and liquid) flowing through the microchannel. Winterton and coauthors [41] introduced the concept of a silicon-based microreactor with isolated thermal zones where nucleation and nanoparticle growth occur. Erdem and coauthors [27] designed a silicon microreactor with Pyrex glass as a microchannel lid for the synthesis of TiO_2_ with different zones heated at different temperatures. All of the quoted examples of high temperature microreactors use external heaters.

During the last two decades, a typical design of integrated heaters in MEMS devices generally, including MEMS microreactors, has made use of deposited high electrical resistance thin films, either polysilicon layers or thin metal films [42]. These may be produced in a variety of ways. As a rule, cleanroom facilities and sophisticated high-end planar technology equipment are needed for that purpose. Tiggelaar and coauthors [43] presented the use of the electron-beam evaporation method for the deposition of a platinum layer heater. Creemer and coauthors [44] described membrane-type microreactors for nanoparticle production with integrated hotplates. To this purpose, the authors used a micro hotplate-based heater made of platinum embedded in a silicon nitride membrane. A 200 nm thick integrated platinum heater deposited by electron-beam evaporation was used to build a microreactor for the synthesis of quantum dots in a submicrometer freestanding nanomembrane, as presented in [45].

Malecha and coauthors described a microreactor with an integrated heater based on low temperature cofired ceramics (LTCC) technology [46]. An integrated microfluidic/thermal platform (high temperature heater) was described in [47]. The microreactor heaters were also fabricated using LTCC technology (gold-based paste was used to deposit heaters). An external heater in a single crystal silicon wafer was described in [48]. An advantage of heaters integrated into single crystalline silicon by diffusion or ion implantation is that they allow higher operating temperatures than the corresponding metallic (Pt) or polysilicon heaters, even allowing temperatures of several hundreds of degrees Celsius.

Some types of microreactors also include the in situ characterization of various parameters. The published works include for instance [49,50,51].

A review of various methods of nanoparticle production using microreactors was published in [52]. Many material and method combinations were presented in the quoted review. Other recent reviews of microreactors can be found in [53,54,55] where the authors describe the design and fabrication of microreactors and their applications.

In this work, we consider two p-type (boron) diffused spiral-shaped heaters monolithically integrated into the Si wafer of a model microreactor fabricated by the well-known method of anodic bonding of silicon with Pyrex glass. The microreactor Pyrex glass lid has a conventional and also well-known microchannel serpentine network isotropically wet etched in it. Our accent was on the new heaters and their heat exchange properties, since we did not observe in literature the use of a similar solution for integrated heaters in microreactors. The main goal was to obtain necessary temperatures in the zones for the nucleation and growth of nanoparticles in a realistic microreactor structure. The temperatures required for the synthesis of TiO_2_ nanoparticles in microreactors range from 60 to 100 °C, depending on the desired size of nanoparticles [27]. As for the model reactor structure itself, it was a wholly completed Si–Pyrex high-temperature microreactor with a serpentine microchannel, however, without full fluidic functionality, but with a fully functional dual heater hot zone. We present here our results on the design, simulation, fabrication and thermal characterization of the two heaters monolithically integrated by boron diffusion into a single crystalline silicon wafer and built into a model high temperature Pyrex–silicon microreactor with a thermal barrier aperture array between them.

## 2. Fabrication

**Figure 1:**
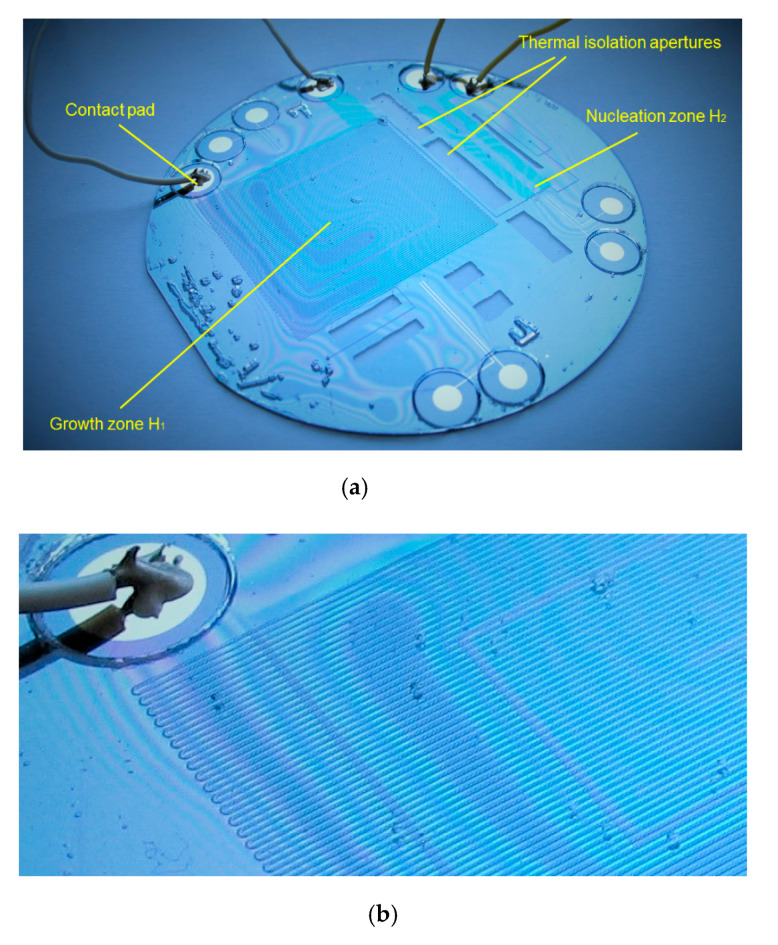
(**a**) Microreactor with integrated p-type diffused heaters in two different zones (the zone for nucleation with a smaller heater H_2_ and the zone for the growth of nanoparticles with a larger heater H_1_) with thermal isolation and metal contact pads and bonding wires; (**b**) enlarged view of the microreactor showing the network of microchannels etched in Pyrex glass, located above the heater.

Figure 1 shows the structure of the fabricated model (dummy) microreactor. One of the two main parts of the microreactor is a silicon wafer bottom into which we diffused acceptor dopant (boron) to fabricate high-resistance p-type heaters. We made two heaters, each in its separate zone. The nucleation of titania nanoparticles should be performed in the first zone, where the smaller heater is integrated, while the second zone, where the larger heater is integrated, is intended for the growth of the nanoparticles. The two zones are separated by apertures in the silicon wafer to prevent conductive heat transfer and thus ensure adequate thermal isolation between the two heaters. As a lid for the Si bottom plate, we used Pyrex glass in which we etched microchannels.

**Figure 2:**
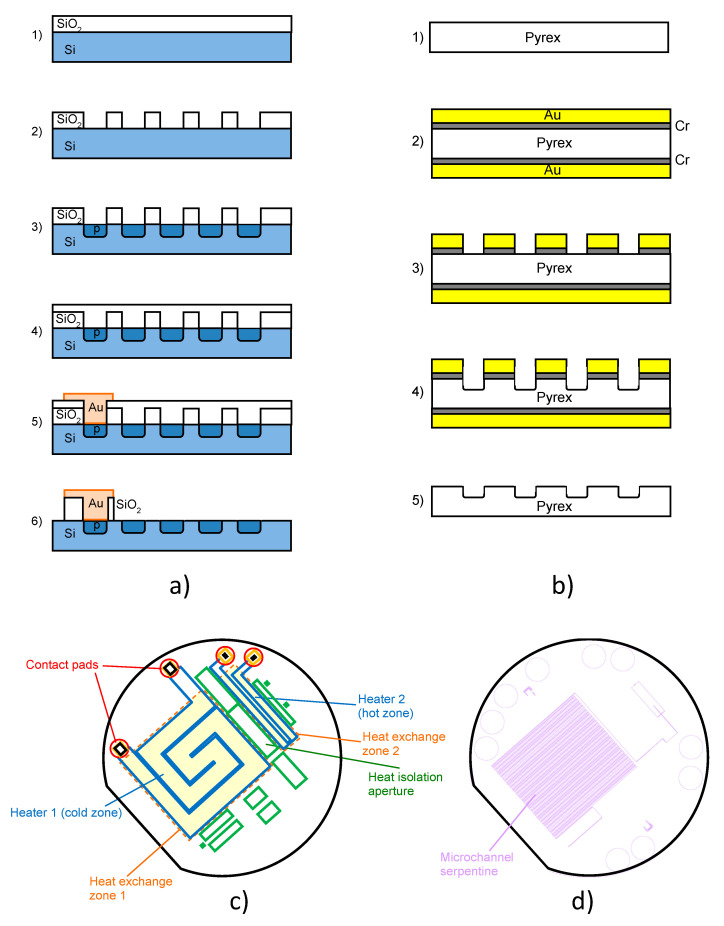
(**a**) Steps for fabrication of p-type Si heater; (**b**) steps for fabrication of microchannel in Pyrex glass; (**c**) photolithographic masks for fabrication of integrated p-type heater in silicon (overlapped, color-coded: blue denotes heaters, green are heat barrier apertures, red are contact pads). The pale yellow fields bounded by dashed orange lines are the heat exchange zones (coinciding with the serpentine microchannel area in Pyrex); (**d**) photolithographic mask defining the meandering microchannel route in Pyrex glass.

In our experiments, we used double side polished, n-type Si wafers of 3 inch diameters as a platform for the integrated heaters. Figure 2a shows the steps for the fabrication of the p-type Si heater. At the beginning, the Si wafer was cleaned and prepared for oxidation of the SiO_2_ layer. The cleaning process was performed with Piranha solution, mixture of sulfuric acid (H_2_SO_4_, 95–98%) and hydrogen peroxide (H_2_O_2_, 30%), with a volume ratio of 3:1 (H_2_SO_4_:H_2_O_2_). SiO_2_ was grown in an oxidation furnace as a masking layer on the Si wafer—Figure 2(a-1). The thermal oxide was obtained at a temperature of 1100 °C. The thickness of masking oxide was 0.6 µm.

**Figure 3:**
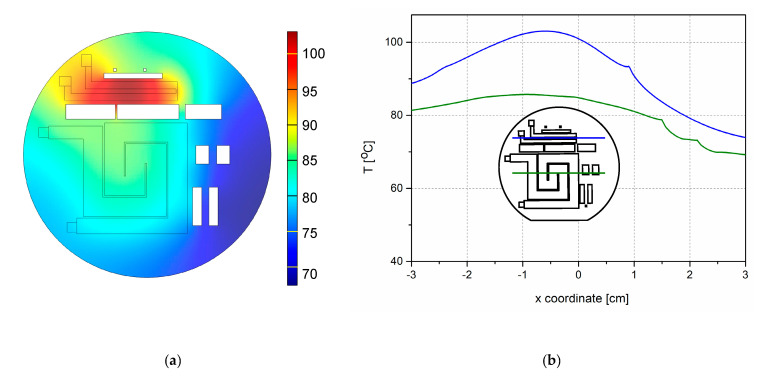
Simulation of temperature distribution on and around the monolithically integrated p-type heaters with the apertures for thermal isolation between the heaters for the current values I_H2_ = 80 mA and I_H1_ = 120 mA after 20 min; (**a**) spatial distribution of temperature; (**b**) temperature gradient across the centers of the both heaters. The inset shows both heaters on the wafer, as well as the thermal isolation apertures (centers are marked by horizontal lines). Green lines denote the larger heater H1, while blue lines correspond to the smaller heater H2.

After the thermal oxidation step, we performed photolithographic patterning for the fabrication of the heater. The SiO_2_ layer was etched in buffered hydrofluoric acid (BHF)—Figure 2(a-2). The second step was p-type diffusion to obtain the heater—Figure 2(a-3). Diffusion of boron was performed at a temperature of 1025 °C for 1 h.

**Figure 4:**
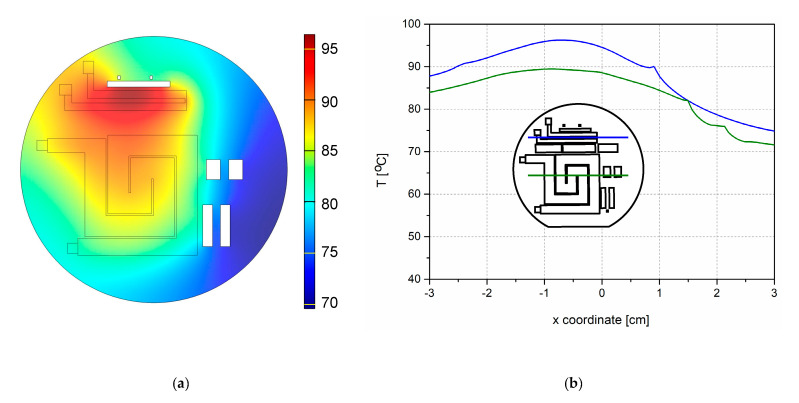
Simulation of temperature distribution in and around the monolithically integrated p-type heaters in the microreactor without thermal isolation between the heaters for the current values I_H2_ = 80 mA and I_H1_ = 120 mA after 20 min; (**a**) spatial distribution of temperature; (**b**) temperature gradient across the centers of both heaters. The inset shows both heaters on the wafer, as well as the thermal isolation apertures (centers are marked by horizontal lines). Green lines denote the larger heater H1, while blue lines correspond to the smaller heater H2.

After the diffusion process, another layer of thermal oxide was fabricated at a temperature of 1100 °C for 25 min—Figure 2(a-4). The thickness of the silicon dioxide layer was about 0.3 µm. The photolithographic process was performed to pattern apertures for thermal isolation and inlets and outlets. This thermal oxide was used as the masking layer for etching the Si wafer in the TMAH (tetramethylammonium hydroxide) water solution. The holes etched throughout silicon were used as thermal isolation for the rest of the microreactor from the heaters as shown on photolithographic masks in Figure 2c.

**Figure 5:**
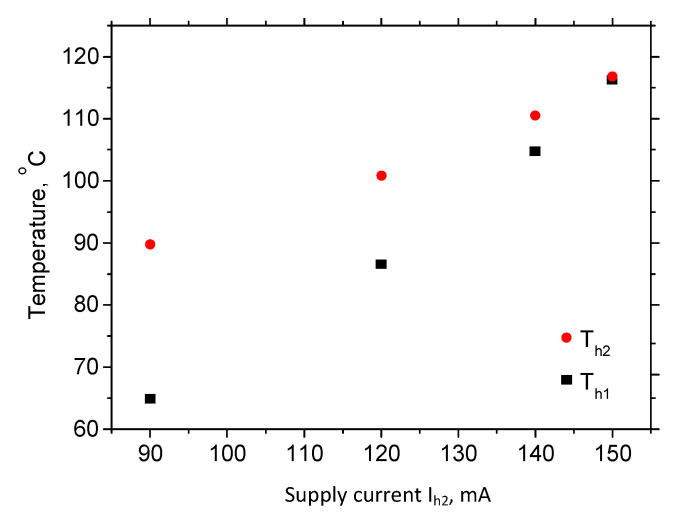
Temperature of both heaters versus the current through the larger heater I_h1_ when the current of the smaller heater I_h2_ is kept constant (80 mA).

The shape of the heaters was spiral, and the measured values of resistance were 270 Ω for the larger heater and 470 Ω for the smaller heater. The contacts were a sputtered thin gold layer with a chromium sublayer—Figure 2(a-5). The sublayer was used to ensure a better adhesion between gold and silicon. The thicknesses of Cr and Au were 10 and 100 nm, respectively.

**Figure 6:**
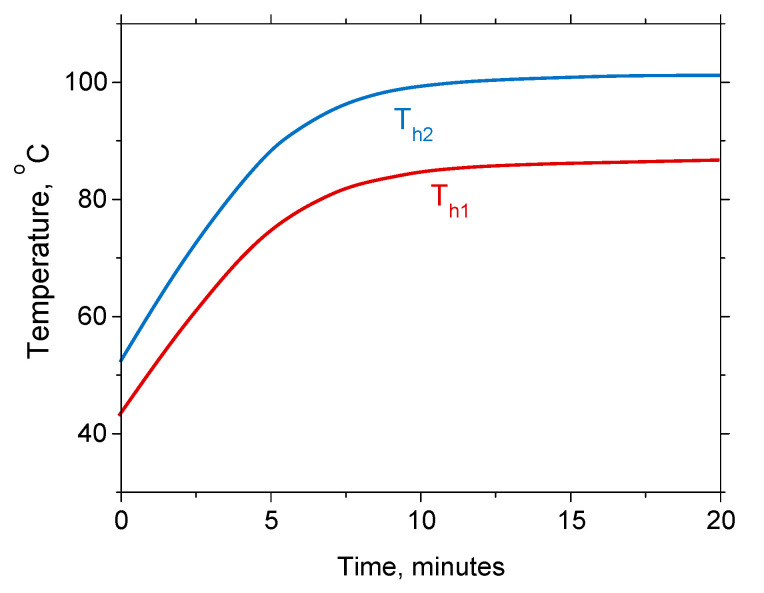
Time evolution of temperature for I_H2_ = 80 mA and I_H1_ = 120 mA during 20 min.

At the end, we etched the SiO_2_ layer to perform anodic bonding between the silicon and the Pyrex glass wafers—Figure 2(a-6). The electric contacts of the microreactor (Figure 1a) were made by silver paste. The microchannel itself was fabricated in the Pyrex glass lid using concentrated HF acid and gold with a sublayer of chromium as the masking layer. The steps for the fabrication of the microchannel in Pyrex glass are shown in Figure 2b. For the microchannels and apertures for metal connections in Pyrex glass, we used asymmetric double-sided isotropic etching. The first step was etching the apertures in Pyrex glass for metal contacts on the bottom side of the wafer. We etched the apertures to a certain depth, the thickness of the residual Pyrex glass on that spot corresponding to the depth of the microchannels located on the top side of Pyrex glass. In the second step, we patterned microchannels with the photolithography process on the top side of the wafer (Figure 2(b-3)), and after that, we simultaneously etched the microchannels (Figure 2(b-4)) and the apertures for metal connections which are on the bottom side of the wafer. The obtained microchannel had a cross-section roughly in the shape of a half-ellipse, their width being along the longer axis of the ellipse. As far as the dimensions are concerned, after isotropic wet etching to obtain channels in Pyrex glass, the obtained channel width was 200 µm, the depth was 75 µm and the total length of the channel was about 3.8 m.

At the end, we etched gold and chromium from the whole wafer (Figure 2(b-5)) to obtain a clear Pyrex glass wafer ready for anodic bonding to the Si wafer. The heater positions overlapped with those of the microchannels.

It can be observed in both Figure 1a,b that the last step, the anodic bonding of the Si wafer to the Pyrex lid, remained incomplete in several locations in this microreactor sample, as visible from the appearance of interference fringes between the silicon wafer and the Pyrex lid. This effectively prevented the microchannels from being used for microfluidic purposes, since it made it impossible to drive the test fluid through them without cross leakage. This obviously disabled any use of the structure for the nanoparticle synthesis. However, all other structural, technological and material details remained intact, enabling us to test the thermal operation of the novel two-zone heater, which was our primal goal at this stage. We denoted this structure as the model microreactor and used it for the “static” heat exchange analysis, i.e., without fluid flow.

Figure 2c shows the set of photolithographic masks for fabrication of the integrated p-type heater. For an easier insight into the roles of separate parts and their relative positions, the masks are shown overlapped, but each in a different color. The spiral heaters are blue, the heat barrier apertures filled with air are green, and the contact pads are red. For an additional insight into the roles of the building blocks, we used pale yellow rectangles bounded by dashed orange lines to present the heat exchange zones. These zones coincide with the microchannel serpentines in Pyrex, shown in Figure 2d, to ensure maximal heat transfer efficiency between the two.

## 3. Results and Discussion

### 3.1. Simulation

We performed finite element method (FEM) simulations of temperature distribution in the heaters. The simulations were made by the Comsol Multiphysics commercial package. We simulated the heat generation due to electric current flow through the heaters, as well as the heat transfer from the heaters. The FEM model uses the Heat Transfer interface of the Heat Transfer module in combination with the Shell, Electric Currents interface from the AC/DC Module. In a steady state, the resistive p-type layer dissipates the generated heat in two ways: on its top side to the Pyrex glass wafer and on its bottom side to the Teflon plate. Both the glass and Teflon plate are cooled by natural convection of the surrounding air. The heat fluxes toward the surroundings were modeled by using heat transfer film coefficients, *h* = 10 W/(m^2^K).

The material parameters for each part of the model were taken from the built-in material database of Comsol Multiphysics, except for the p-type diffused silicon heaters where the data differ from those for pure silicon and where we utilized material parameters from literature [56]. We calculated the values of electrical conductivity for the diffused p-type layer from the measured sheet resistance (≈9 Ω) and the depth of the diffused layer (≈1 μm). The material parameters of importance for our simulations are summarized in Table 1.

We set mesh options to Physics Controlled, and for the element size we chose Normal. For the given model, the mesh generated in such a way consisted of 56,031 domain elements, 17,290 boundary elements and 1404 edge elements.

The problem we solved belongs to multiphysics, and a segregated approach is selected automatically by Comsol Multiphysics, meaning that the problem is subdivided into two segregated steps, each steps representing a single or more physics, and these individual segregated groups are solved sequentially within a single iteration. In our case, the Heat Transfer in Solids Physics was the segregated group 1 (temperature is variable), and both Shell and Electric Current (Shell) Physics were the segregated group 2 (displacement and electric potential are variables).

The solution converged, and after 6 iterations, the errors estimated for the segregated groups were 0.00095 and 1.6 × 10^−16^, respectively. The same results were obtained for Fine and Finer element sizes, 160,350 and 483,442 domain elements, respectively.

We simulated the temperature distribution on the heaters for four sets of applied currents. The current through the smaller heater was kept constant, I_H2_ = 80 mA, and the values of the current through the larger heater, I_H1_, were 90, 120, 140 and 150 mA. Table 2 shows the maximum temperatures on both heaters for all these sets of the applied current values. The set of currents of our interest was 120 and 80 mA for the larger and the smaller heater, respectively. The maximum temperature obtained by simulations for the smaller heater was 103 °C, and the maximum temperature for the larger heater was 86 °C—Figure 3. Obviously, the higher temperature of the smaller heater was a consequence of its higher Ohmic resistance.

Figure 3a shows the simulated temperature distribution on heaters with thermal isolation between them. The uniformity of the temperature on the surface of the heaters was satisfactory (Figure 4b). Figure 4a shows the simulated temperature distribution on the heaters without thermal isolation between them for the same applied current. We see that the temperature on the surface of the heaters without isolation differed significantly from that on the surface of the heaters with thermal isolation. The temperature maximum on the smaller heater was lower in the case without thermal isolation, and the maximum temperature of the larger heater was higher—Figure 4a,b. We conclude that the thermal isolation plays a significant role in temperature distribution between the heaters and across the whole microreactor.

### 3.2. Experimental Results

We performed temperature measurements for the same sets of applied currents we used in our simulations. To supply a constant current to obtain the required temperature, we connected the contact pads of the heaters to the dual output DC power supply Agilent E3466A. Temperature, together with the solution concentration and the retention time, obviously represents one of the key parameters for nanoparticle synthesis in every high-temperature microreactor. The shapes and the sizes of nanoparticles are dependent on the synthesis temperature. The first zone, with the smaller heater, is used for nucleation of nanoparticles and the second, with the larger heater, for nanoparticle growth. In this work, we applied different values of current on both heaters to simultaneously obtain different values of synthesis temperatures. For our future investigations, we need nanoparticles with sizes between 20 and 30 nm, and the temperatures for these nanoparticle sizes are 100 °C on the smaller heater and 80 °C on the larger heater [27]. To determine the spatial distribution of temperature, we used an infrared (IR) camera FLIR 660. From the thermal images, we read temperatures across the central line of each heater. The microreactor was mounted on a Teflon chuck to improve its thermal isolation.

We measured temperature for four sets of currents, as shown in Table 3. We applied a constant current I_H2_ of 80 mA to the smaller heater, while we varied the current I_H1_ from 90 to 150 mA on the larger heater. Measurements were performed every five minutes during 20 min. Figure 5 shows temperatures at both the heaters in the twentieth minute versus the applied current. The temperatures of the both heaters rose with the increase in the current I_H1_. The temperature of the larger (cooler) heater increased, as observed in Figure 5, even though only the current of the smaller (hotter) heater was varied, which points to a less than satisfactory temperature isolation between the two heaters in spite of the existence of the barrier apertures. Thus, additional external cooling appears necessary to pinpoint the temperature difference between the hotter and cooler zone. This could be performed passively by a fan or actively, by e.g., a thermoelectric cooler.

The presented analysis regards the case without a fluid flow through the microchannel. In the case with an established fluid flow, we could expect that the fluid itself will dissipate at least a part of the generated heat and thus improve the efficiency of the heat removal. This was the reason why the heaters were simulated and supplied to generate somewhat higher temperatures without fluid flow. In our further experiments with a fully functional microreactor with an established nominal fluid flow instead of the model structure without it, we intend to further investigate this issue.

Our simulations and measurements show that the currents of 120 and 80 mA through the larger and the smaller heater, respectively, provide temperatures necessary for a successful synthesis of TiO_2_ nanoparticles. The time evolution of the heater temperature for the quoted current values is shown in Figure 6.

**Figure 7:**
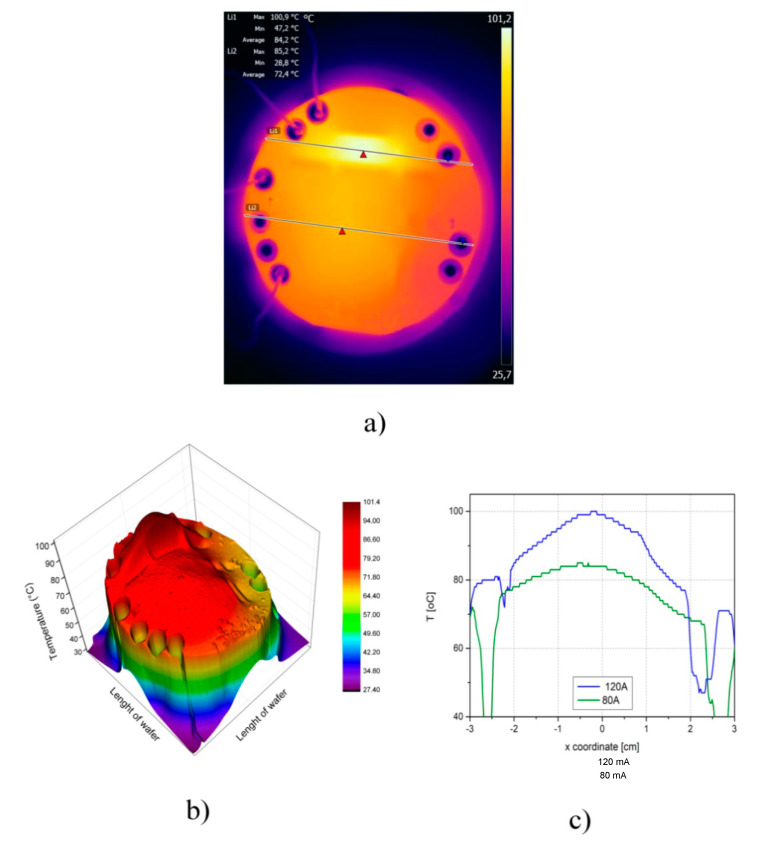
Temperature measurements for monolithically integrated p-type heaters in the microreactor for the current values I_H2_ = 80 mA and I_H1_ = 120 mA after 20 min; (**a**) IR thermal image of temperature distribution on the surface of the Si wafer; (**b**) 3D image of the temperature distribution across the Si wafer; (**c**) temperature distribution across the centers of both heaters.

We see that the temperatures of the heaters H_1_ and H_2_ increased during the first 10 min, at which point the temperatures of the both heaters became constant. The maximum temperatures of the heaters were 101.2 °C for H_2_ and 86.6 °C for H_1_. We achieved a satisfactory agreement between the simulations and the experimental results. The thermal image in Figure 7a shows that the temperature distribution maximum for both heaters was in the area important for the synthesis of nanoparticles. We conclude that the temperature distribution was satisfactory for the production of titania nanoparticles in our microreactor. The experimental results also show that the thermal isolation significantly influenced the surface temperature distribution of both the monolithically integrated heaters. Figure 7b shows again the role and importance of the thermal isolation between the heaters. Finally, Figure 7c shows the temperature distribution through the centers of both heaters.

## 4. Conclusions and Future Works for Verification of the Design and Results

In this paper, we presented design, simulation, fabrication and characterization of novel p-type boron diffused monolithically integrated heaters in the single crystalline silicon wafer of a model high-temperature microreactor for the synthesis of TiO_2_ nanoparticles. Two p^+^ heaters with a spiral shape were diffused in a silicon wafer and were thermally separated by an array of air-filled apertures. In this way, two different temperature zones were simultaneously obtained in a single microreactor. The well-known silicon–Pyrex glass anodically bonded structures with a serpentine microchannel were used. A semifunctional microreactor was utilized for the heat exchange analysis, with a proper design and with full thermal functionality, but without a microfluidic performance. Static characterization of the heaters was performed using electric characterization and infrared thermal imaging. A homogenous temperature profile across the heaters was obtained, which was in good agreement with the results of finite element simulation. For the sake of quantitative comparison, for the required values of temperature in the cold (H1) and hot (H2) zone of 80 °C and 100 °C, respectively (fluid regularly flowing through the microchannel), we obtained numerically simulated data of 86 and 103 °C (no fluid flow), while we experimentally measured 86.6 and 101.2 °C (no fluid flow). The fluid flow should act as a coolant and decrease the temperature somewhat. Thus, the obtained temperatures roughly satisfy the requirements for two temperature zone microreactor synthesis of TiO_2_ nanoparticles.

As far as we are aware, these are the first monolithically integrated diffused single crystalline heaters in a microreactor. Compared to other types of heaters typically used for the same purpose (metallic, most often platinum, or polysilicon), they allow higher operating temperatures (according to the literature data). The advantages of monolithic integration should include an improved mechanical and thermal integration of the heater with the rest of the silicon wafer; while at the same time, it should enable us to reach accurate control over the heater parameters through the well-known spatial profiling of boron diffusion.

Here, we demonstrate proof of concept works on experimental “dry testing” of the diffused two-zone heaters integrated with a model microreactor—i.e., a full electrical and thermal characterization of the heaters’ performance, but without fluid flow through the microchannels. However, since the flow will modify the temperature distribution within the microreactor, our immediate plan is to use the experimental setup identical to the one used here to fabricate microreactors fully functional in the microfluidic sense and perform experiments with the fluid flow through serpentine microchannels, while at the same inspecting heat exchange in our novel diffused two-zone heaters and within the microreactor as a whole.

We also intend to enhance the heat dissipation of the device by implementing a fan system, or, if proven unsatisfactory, by incorporating a thermoelectric cooler to improve the heat exchange control. We also intend to experimentally investigate the maximum achievable operating temperatures in our microreactor system. In this way, we intend to check the literature claims as regards the main advantage of the boron-diffused heaters over the conventional Si/Pyrex microreactors with a thermal resistance module directly connected to the silicon side, namely the operation at higher operating temperatures.

Our immediate next steps after replacing a model reactor with a corresponding fully functional reactor equipped with the novel type of diffused two-zone heater will obviously include the synthesis and characterization of titania nanoparticles with controlled parameters, which should serve as a definitive validation of our approach.

By adjusting the supply currents, a wide range of other temperatures can be achieved. Thus, the same setup can be used to fabricate nanoparticles of different other materials with various parameters and geometries. Our method can be generally applied in other MEMS and MOEMS (Micro-Opto-Electro-Mechanical Systems) that include high temperature processes. Possible generalizations could include a system where the number of temperature zones is larger than two, or even a graded temperature change across the microreactor. Complex systems, such as e.g., labs on a chip, as well as those that incorporate temperature-actuated micromachines, could also profit from the described approach.

## Figures and Tables

**Table 1:** Material parameters used in our numerical simulations.

Material	Thermal Conductivity [W/mK]	Heat Capacity at Constant Pressure [J/kgK]	Electrical Conductivity [S/m]
Pure silicon	131	700	Approx. 0
p^+^ silicon (heaters)	75	750	120,000
Gold	317	129	45.6 × 10^6^
Silica	1.4	730	Approx. 0

**Table 2:** Simulated maximum temperatures on the smaller heater H_1_ and the larger heater H_2._

Variable	I_H1_ (mA)	I_H2_ (mA)	I_H1_ (mA)	I_H2_ (mA)	I_H1_ (mA)	I_H2_ (mA)	I_H1_ (mA)	I_H2_ (mA)
Current (mA)	90	80	120	80	140	80	150	80
Temperatures (°C)	68.5	93	86	103	104	113	114.3	119.6

**Table 3:** Experimentally measured maximum temperatures on the heaters H_1_ and H_2._

Variable	I_H1_ (mA)	I_H2_ (mA)	I_H1_ (mA)	I_H2_ (mA)	I_H1_ (mA)	I_H2_ (mA)	I_H1_ (mA)	I_H2_ (mA)
Current (mA)	90	80	120	80	140	80	150	80
Temperatures (°C)	64.8	89.8	86.6	101.2	104.8	110.7	116.3	116.9

## References

[1] Pohar A., Lakner M., Plazl I. (2012). Parallel flow of immiscible liquids in a microreactor: Modeling and experimental study. Microfluid. Nanofluidics.

[2] Beebe D.J., Mensing G.A., Walker G.M. (2002). Physics and applications of microfluidics in biology. Annu. Rev. Biomed. Eng..

[3] Lagally E.T., Mathies R.A. (2004). Integrated genetic analysis microsystems. J. Phys. D Appl. Phys..

[4] Nielsen C.A., Chrisman R.W., LaPointe R.E., Miller T.E. (2002). Novel tubing microreactor for monitoring chemical reactions. Anal. Chem..

[5] DeWitt S.H. (1999). Micro reactors for chemical synthesis. Curr. Opin. Chem. Biol..

[6] Jia H., Wong Y.L., Jian A., Tsoi C.C., Wang M., Li W., Zhang W., Sang S., Zhang X. (2019). Microfluidic Reactors for Plasmonic Photocatalysis Using Gold Nanoparticles. Micromachines.

[7] Yoshida J.I., Nagaki A., Yamada T. (2008). Flash chemistry: Fast chemical synthesis by using microreactors. Chem. Eur. J..

[8] Zhao C.-X., He L., Qiao S.Z., Middelberg A.P. (2011). Nanoparticle synthesis in microreactors. Chem. Eng. Sci..

[9] Wiles C., Watts P. (2011). Recent advances in micro reaction technology. Chem. Commun..

[10] Yue J., Falke F.H., Schouten J.C., Nijhuis T.A. (2013). Microreactors with integrated UV/Vis spectroscopic detection for online process analysis under segmented flow. Lab Chip.

[11] Ehgartner J., Sulzer P., Burger T., Kasjanow A., Bouwes D., Krühne U., Klimant I., Mayr T. (2016). Online analysis of oxygen inside silicon-glass microreactors with integrated optical sensors. Sens. Actuators B Chem..

[12] Mensinger H., Richter T., Hessel V., Döpper J., Ehrfeld W. (1995). Microreactor with Integrated Static Mixer and Analysis System. Micro Total Analysis Systems.

[13] Pfeiffer S.A., Borisov S.M., Nagl S. (2017). In-line monitoring of pH and oxygen during enzymatic reactions in off-the-shelf all-glass microreactors using integrated luminescent microsensors. Microchim. Acta.

[14] Abgrall P., Gue A. (2007). Lab-on-chip technologies: Making a microfluidic network and coupling it into a complete microsystem—A review. J. Micromech. Microeng..

[15] Reyes D.R., Iossifidis D., Auroux P.-A., Manz A. (2002). Micro total analysis systems. 1. Introduction, theory, and technology. Anal. Chem..

[16] Srinivas S., Dhingra A., Im H., Gulari E. (2004). A scalable silicon microreactor for preferential CO oxidation: Performance comparison with a tubular packed-bed microreactor. Appl. Catal. A General.

[17] De La Iglesia O., Sebastián V., Mallada R., Nikolaidis G., Coronas J., Kolb G., Zapf R., Hessel V., Santamaría J. (2007). Preparation of Pt/ZSM-5 films on stainless steel microreactors. Catal. Today.

[18] Knitter R., Göhring D., Risthaus P., Hausselt J. (2001). Microfabrication of ceramic microreactors. Microsyst. Technol..

[19] Rašljić M., Građanski I., Smiljanić M.M., Janković N.Z., Lazić Ž., Cvetanović Zobenica K. Microfabrication of Bifurcated Microchannels with PDMS and ABS. Proceedings of the 4th International Conference on Electrical, Electronics and Computing Engineering.

[20] Yousuff C.M., Danish M., Ho E.T.W., Kamal Basha I.H., Hamid N.H.B. (2017). Study on the optimum cutting parameters of an aluminum mold for effective bonding strength of a PDMS microfluidic device. Micromachines.

[21] Marre S., Adamo A., Basak S., Aymonier C., Jensen K.F. (2010). Design and Packaging of Microreactors for High Pressure and High Temperature Applications. Ind. Eng. Chem. Res..

[22] Kusakabe K., Miyagawa D., Gu Y., Maeda H., Morooka S. (2001). Development of self-heating microreactor for catalytic reactions. J. Chem. Eng. Jpn..

[23] Ari J., Louvet G., Ledemi Y., Célarié F., Morais S., Bureau B., Marre S., Nazabal V., Messaddeq Y. (2020). Anodic bonding of mid-infrared transparent germanate glasses for high pressure-high temperature microfluidic applications. Sci. Technol. Adv. Mater..

[24] Shanks H., Maycock P., Sidles P., Danielson G. (1963). Thermal conductivity of silicon from 300 to 1400 K. Phys. Rev..

[25] Ibach H. (1969). Thermal expansion of silicon and zinc oxide (I). Phys. Status Solidi.

[26] Yang J., Liu Y., Rauch C.B., Stevens R.L., Liu R.H., Lenigk R., Grodzinski P. (2002). High sensitivity PCR assay in plastic micro reactors. Lab Chip.

[27] Erdem E.Y., Cheng J.C., Doyle F.M., Pisano A.P. (2014). Multi-Temperature Zone, Droplet-based Microreactor for Increased Temperature Control in Nanoparticle Synthesis. Small.

[28] Wang J., Bai S., Wang Y., Wang T., Luo G. (2019). Continuous and ultrafast preparation of In(OH)_3_, InOOH, and In_2_O_3_ series in a microreactor for gas sensors. Industr. Eng. Chem. Res..

[29] Wagner J., Köhler J. (2005). Continuous synthesis of gold nanoparticles in a microreactor. Nano Lett..

[30] Haider A.J., AL–Anbari R.H., Kadhim G.R., Salame C.T. (2017). Exploring potential environmental applications of TiO_2_ nanoparticles. Energy Procedia.

[31] Paz Y. (2010). Application of TiO_2_ photocatalysis for air treatment: Patents’ overview. Appl. Catal. B Environ..

[32] Mor G.K., Shankar K., Paulose M., Varghese O.K., Grimes C.A. (2006). Use of highly-ordered TiO_2_ nanotube arrays in dye-sensitized solar cells. Nano Lett..

[33] Abdeslam A., Fouad K., Khalifa A. (2020). Design and optimization of platinium heaters for gas sensor applications. Dig. J. Nanomater. Biostruct..

[34] Liu Q., Ding G., Wang Y., Yao J. (2018). Thermal performance of micro hotplates with novel shapes based on single-layer SiO_2_ suspended film. Micromachines.

[35] Quiram D.J., Jensen K.F., Schmidt M.A., Mills P.L., Ryley J.F., Wetzel M.D., Kraus D.J. (2007). Integrated microreactor system for gas-phase reactions. Micro Instrumentation for High Throughput Experimentation and Process Intensification—A Tool for PAT.

[36] Tommasi A., Cocuzza M., Perrone D., Pirri C.F., Mosca R., Villani M., Delmonte N., Zappettini A., Calestani D., Marasso S.L. (2017). Modeling, fabrication and testing of a customizable micromachined hotplate for sensor applications. Sensors.

[37] Resnik D., Vrtačnik D., Možek M., Pečar B., Amon S. (2011). Experimental study of heat-treated thin film Ti/Pt heater and temperature sensor properties on a Si microfluidic platform. J. Micromech. Microeng..

[38] Rašljić M., Smiljanić M.M., Lazić Ž., Radulović K., Zobenica K.C., Radović D.V. Two types of integrated heaters for synthesis of TiO2 nanoparticles in microreactors. Proceedings of the 5th International Conference on Electrical, Electronic and Computing Engineering.

[39] Chan E.M., Mathies R.A., Alivisatos A.P. (2003). Size-controlled growth of CdSe nanocrystals in microfluidic reactors. Nano Lett..

[40] Yen B.K., Günther A., Schmidt M.A., Jensen K.F., Bawendi M.G. (2005). A microfabricated gas–liquid segmented flow reactor for high-temperature synthesis: The case of CdSe quantum dots. Angew. Chem..

[41] Winterton J.D., Myers D.R., Lippmann J.M., Pisano A.P., Doyle F.M. (2008). A novel continuous microfluidic reactor design for the controlled production of high-quality semiconductor nanocrystals. J. Nanopart. Res..

[42] Bayt R.L., Breuer K.S. (2001). A silicon heat exchanger with integrated intrinsic-point heater demonstrated in a micropropulsion application. Sens. Actuators.

[43] Tiggelaar R.M., Van Male P., Berenschot J., Gardeniers J., Oosterbroek R., De Croon M., Schouten J., van den Berg A., Elwenspoek M.C. (2005). Fabrication of a high-temperature microreactor with integrated heater and sensor patterns on an ultrathin silicon membrane. Sens. Actuators A Phys..

[44] Creemer J.F., Helveg S., Kooyman P.J., Molenbroek A.M., Zandbergen H.W., Sarro P.M. (2010). A MEMS reactor for atomic-scale microscopy of nanomaterials under industrially relevant conditions. J. Microelectromech. Syst..

[45] Nightingale A.M., de Mello J.C. (2010). Microscale synthesis of quantum dots. J. Mater. Chem..

[46] Malecha K., Pijanowska D.G., Golonka L.J., Torbicz W. (2009). LTCC microreactor for urea determination in biological fluids. Sens. Actuators B Chem..

[47] Martínez-Cisneros C.S., Gómez-de Pedro S., Puyol M., García-García J., Alonso-Chamarro J. (2012). Design, fabrication and characterization of microreactors for high temperature syntheses. Chem. Eng. J..

[48] Mihailović M., Creemer J., Sarro P. Monocrystalline Si-based microhotplate heater. Proceedings of the SAFE/STW.

[49] Beuvier T., Panduro E.A.C., Kwaśniewski P., Marre S., Lecoutre C., Garrabos Y., Aymonier C., Calvignac B., Gibaud A. (2015). Implementation of in situ SAXS/WAXS characterization into silicon/glass microreactors. Lab Chip.

[50] Tofighi G., Lichtenberg H., Pesek J., Sheppard T.L., Wang W., Schöttner L., Rinke G., Dittmeyer R., Grunwaldt J.D. (2017). Continuous microfluidic synthesis of colloidal ultrasmall gold nanoparticles: In situ study of the early reaction stages and application for catalysis. React. Chem. Eng..

[51] Cao E., Brett G., Miedziak P.J., Douthwaite J.M., Barrass S., McMillan P.F., Hutchings G.J., Gavriilidis A. (2017). A micropacked-bed multi-reactor system with in situ raman analysis for catalyst evaluation. Catal. Today.

[52] Solsona M., Vollenbroek J., Tregouet C., Nieuwelink A.-E., Olthuis W., Van Den Berg A., Weckhuysen B., Odijk M. (2019). Microfluidics and catalyst particles. Lab Chip.

[53] Bojang A.A., Wu H.-S. (2020). Design, Fundamental Principles of Fabrication, and Applications of Microreactors. Processes.

[54] Suryawanshi P.L., Gumfekar S.P., Bhanvase B.A., Sonawane S.H., Pimplapure M.S. (2018). A review on microreactors: Reactor fabrication, design, and cutting-edge applications. Chem. Eng. Sci..

[55] Yue J. (2018). Multiphase flow processing in microreactors combined with heterogeneous catalysis for efficient and sustainable chemical synthesis. Catal. Today.

[56] Ohishi Y., Xie J., Miyazaki Y., Aikebaier Y., Muta H., Kurosaki K., Yamanaka S., Uchida N., Tada T. (2015). Thermoelectric properties of heavily boron-and phosphorus-doped silicon. Jpn. J. Appl. Phys..

